# DNA microarray‐based analysis of voluntary resistance wheel running reveals novel transcriptome leading robust hippocampal plasticity

**DOI:** 10.14814/phy2.12206

**Published:** 2014-11-20

**Authors:** Min Chul Lee, Randeep Rakwal, Junko Shibato, Koshiro Inoue, Hyukki Chang, Hideaki Soya

**Affiliations:** 1 Laboratory of Exercise Biochemistry and Neuroendocrinology, Faculty of Health and Sport Sciences, University of Tsukuba, TsukubaIbaraki, Japan; 2 International Research Fellow of the Japan Society for the Promotion of Science, Tokyo, Japan; 3 Organization for Educational Initiatives, University of Tsukuba, TsukubaIbaraki, Japan; 4 Department of Anatomy, Showa University School of Medicine, ShinagawaTokyo, Japan; 5 Human Movement Science, College of Natural Science, Seoul Women's University, Nowon‐guSeoul, Korea

**Keywords:** DNA microarray, hippocampus, inflammatory cytokine, resistance wheel running

## Abstract

In two separate experiments, voluntary resistance wheel running with 30% of body weight (RWR), rather than wheel running (WR), led to greater enhancements, including adult hippocampal neurogenesis and cognitive functions, in conjunction with hippocampal brain‐derived neurotrophic factor (BDNF) signaling (Lee et al., J Appl Physiol, 2012; Neurosci Lett., 2013). Here we aimed to unravel novel molecular factors and gain insight into underlying molecular mechanisms for RWR‐enhanced hippocampal functions; a high‐throughput whole‐genome DNA microarray approach was applied to rats performing voluntary running for 4 weeks. RWR rats showed a significant decrease in average running distances although average work levels increased immensely, by about 11‐fold compared to WR, resulting in muscular adaptation for the fast‐twitch plantaris muscle. Global transcriptome profiling analysis identified 128 (sedentary × WR) and 169 (sedentary × RWR) up‐regulated (>1.5‐fold change), and 97 (sedentary × WR) and 468 (sedentary × RWR) down‐regulated (<0.75‐fold change) genes. Functional categorization using both pathway‐ or specific‐disease‐state‐focused gene classifications and Ingenuity Pathway Analysis (IPA) revealed expression pattern changes in the major categories of disease and disorders, molecular functions, and physiological system development and function. Genes specifically regulated with RWR include the newly identified factors of *NFATc1, AVPR1A,* and *FGFR4,* as well as previously known factors, *BDNF* and *CREB mRNA*. Interestingly, RWR down‐regulated multiple inflammatory cytokines (*IL1B, IL2RA,* and *TNF*) and chemokines (*CXCL1, CXCL10, CCL2,* and *CCR4*) with the *SYCP3*,* PRL* genes, which are potentially involved in regulating hippocampal neuroplastic changes. These results provide understanding of the voluntary‐RWR‐related hippocampal transcriptome, which will open a window to the underlying mechanisms of the positive effects of exercise, with therapeutic value for enhancing hippocampal functions.

## Abstract (precis)

New information on the voluntary RWR influenced transcriptome in rat hippocampus. Selected gene candidates may be a critical role in the development of hippocampal adaptations in RWR.

## Introduction

Physical activity, such as exercise is beneficial for brain health and function. In the brain, the hippocampus is the region that is thought to play a crucial role in enhancing cognitive functions, including learning and memory (Fordyce and Wehner 1993; Gould et al. 1999; van Praag et al. 1999). A number of studies have shown that exercise enhances spatial learning and memory in animals (Samorajski et al. 1985; Kempermann et al. 2004; Vaynman et al. 2004). In rodents, running is also known to enhance adult neurogenesis in the dentate gyrus and the magnitude of hippocampal long‐term potentiation (van Praag et al. 1999; Rhodes et al. 2003), which is associated with hippocampus‐dependent cognitive functions (Farmer et al. 2004; Vaynman et al. 2004).

Previous research suggests that exercise‐induced positive effects are in part probably due to an increased expression of the hippocampal brain‐derived neurotrophic factor (BDNF) (Vaynman et al. 2004; Adlard et al. 2005). BDNF is an important protein in supporting the growth, development and survival of neurons as well as in synaptic plasticity and certain types of learning and memory (Hofer et al. 1990; Korte et al. 1995). Exercise increases hippocampal BDNF mRNA and protein expression levels (Neeper et al. 1995; Soya et al. 2007), which in turn promotes adult neurogenesis (Pencea et al. 2001; Lee et al. 2002). Alteration in BDNF signaling is actually required in order to allow the effects of exercise on hippocampal plasticity in rodents: blocking BDNF signaling prevents exercise‐induced gain in learning and memory (Vaynman et al. 2004) and neurogenesis (Li et al. 2008). This may suggest that “more BDNF is better” for the enhancement of hippocampal functions. Despite its importance in brain function and health, it is highly unlikely that BDNF works alone, implying that a search for novel factors is in order.

Exercise has two aspects: amount and intensity. In running, it may not always be the case that the amount of exercise, i.e. running distance, is important for adaptation. Rather, running intensity might also be a critical factor. Most studies supporting the beneficial effects of exercise focus on the amount of exercise, not the exercise intensity, which may be another potentially important, and still uninvestigated, factor in exercise‐influenced brain adaptations. This also means that the optimal exercise condition for generating such beneficial effects on hippocampal plasticity remains contentious.

Recently, we examined hippocampal neurogenesis and cognitive functions in the adult rat hippocampus after 4 weeks of voluntary wheel running with and without a load (Lee et al. 2012, 2013). We found that the average work levels significantly increased and the average running distance decreased to about half in the voluntary resistance exercise with 30% of body weight group (RWR) compared to free wheel running without a load group (WR). This in turn elicited muscular adaptation for fast‐twitch plantaris muscle without causing any negative stress effects. Furthermore, both RWR and WR improved cognitive functions as well as increased expression of hippocampal BDNF signaling. That previous study demonstrated for the first time that voluntary resistance exercise might be an effective model for enhancing spatial learning and memory associated with increased hippocampal BDNF signaling, even with short distances (Lee et al. 2012). The fact that RWR exercise showed shorter distance but hyper‐work levels, led us to hypothesize that, “less is more” for effective running distances leading to hippocampal plasticity. However, the underlying molecular mechanisms and factors behind this enhancement, other than BDNF, remain unknown.

Therefore, we took our study to the next level and, using data from the same sample pool, performed a search for the RWR condition‐influenced hippocampal transcriptome using a whole‐genome DNA microarray chip (DeRisi et al. 1997). Although high‐throughput omics approaches, including transcriptome profiling, can be helpful in providing detailed insight into molecular factors underlying cell, tissue and organism level biology, relatively few studies exist in respect to exercise‐induced neuronal plasticity and brain function (Tong et al. 2001; Molteni et al. 2002; Hunsberger et al. 2007; Stranahan et al. 2007; Kohman et al. 2011). Moreover, there are also no studies that have examined the effect of RWR conditions on the rat hippocampus transcriptome. Thus, building upon the knowledge obtained from our previous research (Lee et al. 2012, 2013), here we aim to expand our understanding of the RWR exercise model at the level of the transcriptome.

It was our hypothesis that RWR, short distances but high work levels, would generate changes at the level of transcription within the hippocampus and have a unique gene expression profile compared to WR. The obtained global gene expression data provided an answer: we unraveled novel gene expressions, especially with RWR, where surprisingly the dominant expression change was down‐expression. Based on the gene inventory and bioinformatics analyses, we have postulated underlying molecular mechanisms behind RWR in developing robust hippocampal functions.

## Materials and Methods

### Animals

Ten‐week‐old male Wistar rats (320–340 g) were obtained from SEAS Co., Ltd. (Saitama, Japan) and randomly allocated to three groups as follows: (1) housed in standard cages and used as nonactive controls (sedentary, *n *=**10); (2) wheel running with no resistance (WR, *n *=**8); and (3) housed in cages with resistance running wheels with an adjustable resistance (RWR, *n *=**8). All rats were individually housed and kept in a controlled environment with a 12/12 h light/dark cycle (lights on at 8:00 am) and given ad libitum access to food and water. All the experiments were performed in accordance with protocols approved by the University of Tsukuba Animal Experiment Committee, based on the NIH Guidelines for the Care and Use of Laboratory Animals (NIH publication, 1996).

### Running‐wheel apparatus and loading protocol

This RWR apparatus and its setup for resistance running were performed as described previously (Ishihara et al. 1998; Lee et al. 2012). Briefly, the rats of both running wheel groups (WR and RWR) had free access to a specially designed running wheel apparatus (diameter = 31.8 cm, width = 10 cm; Rat Analyzer KI‐103, Aptec Inc., Kyoto, Japan) for 24 h/day. The resistance attached to the wheel could be changed with a range of 0 to 200 g. The rats in the RWR group were exercised with minimum resistance (i.e., 4.5 g) for the first week, and then the resistance was progressively increased to reach 30% of their body weight during the 4 weeks of exercise. Daily work levels as total energy expenditure during exercise were calculated and expressed relative to body weight and day as follows: Work (J) = Force (N) × Distance (m) / Body weight (kg) / day where force is the resistance of the wheel and distance is the number of revolutions times the circumference of the wheel.

### Sample collection and total RNA extraction

**Figure 1.**
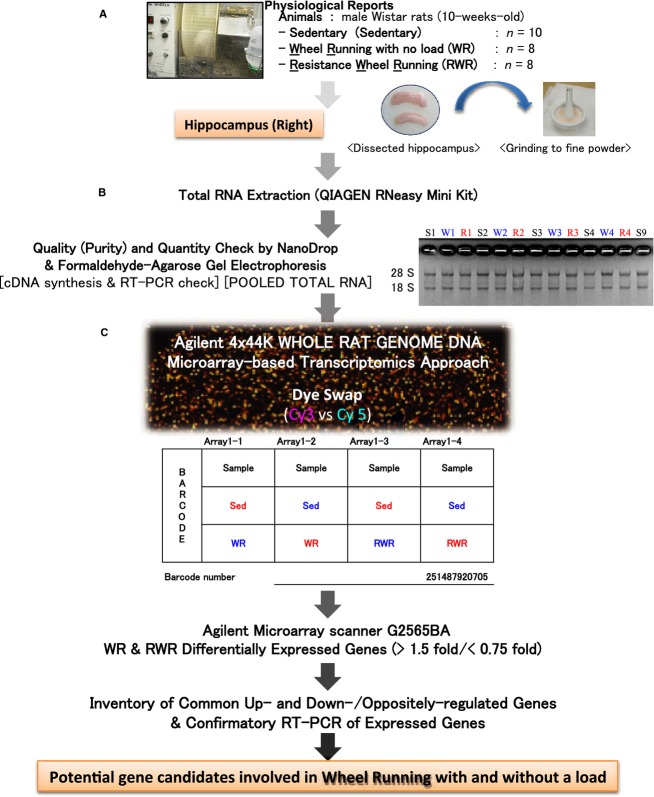
Workflow from sampling and grinding of the hippocampus, total RNA extraction, and DNA microarray analysis of the hippocampus. (A) The rats hippocampi were rapidly dissected and ground to a fine powder in liquid nitrogen and stored at –80°C. (B) Total RNA extraction from the finely powdered hippocampus. Total RNA quality was confirmed by both spectrophotometry and agarose‐gel electrophoresis. (C) DNA microarray chip showing the hybridized sample combinations (Sed × WR and Sed × RWR) and dye‐swap (Cy3 vs. Cy5).

Between 08:00 and 12:00 on the day after the final day of exercise, the animals were decapitated using a guillotine. To measure the physiological states of the rats, adrenal glands, thymus, soleus, and plantaris muscles were sampled and weighed (wet weight) immediately after decapitation. The hippocampi were also rapidly dissected, snap frozen in liquid nitrogen, and stored at −80°C prior to further analysis. The deep‐frozen hippocampi were transferred to a precooled (in liquid nitrogen) mortar and ground with a pestle to a very fine powder with liquid nitrogen. The workflow for preparation of fine tissue powders for hippocampal gene analysis is given in Fig. 1A. The powdered samples were transferred to 2 mL Eppendorf microtubes and stored in aliquots at −80°C until used for extraction of total RNA. Total RNA was extracted from ~50 mg sample powder using the QIAGEN RNeasy Mini Kit (QIAGEN, Germantown, MD). To verify the quality of this RNA, the yield and purity were determined spectrophotometrically (IMPLEN, Germany) and visually confirmed using formaldehyde‐agarose gel electrophoresis (Fig. 1B).

### cDNA synthesis and semi‐quantitative RT‐PCR

To validate the total RNA quality and subsequently synthesized cDNA, RT‐PCR was performed. As a validated endogenous control, glyceraldehyde 3‐phosphate dehydrogenase (GAPDH) and β‐actin was amplified in a separate reaction for normalization. Briefly, total RNA samples were first DNase‐treated with RNase free DNase (Stratagene; Agilent Technologies, La Jolla, CA). First strand cDNA was then synthesized in a 20 *μ*L reaction mixture with an AffinityScript QPCR cDNA Synthesis Kit (Stratagene; Agilent Technologies) according to the protocol provided by the manufacturer, using 1 *μ*g total RNA isolated from each control and treated hippocampi sample. The reaction conditions were: 25°C for 5 min, 42°C for 5 min, 55°C for 40 min and 95°C for 5 min. The synthesized cDNA was made up to a volume of 50 *μ*L with sterile water supplied in the kit. The reaction mixture contained 0.6 *μ*L of the first‐strand cDNA, 7 pmols of each primer set and 6.0 *μ*L of the Emerald Amp PCR Master Mix (2× premix) (TaKaRa Shuzo, Shiga, Japan) in a total volume of 12 *μ*L. Thermal‐cycling (Applied Biosystems, Tokyo, Japan) parameters were as follows: after an initial denaturation at 97°C for 5 min, samples were subjected to a cycling regime of 20–40 cycles at 95°C for 45 sec, 55°C for 45 sec and 72°C for 1 min. At the end of the final cycle, an additional extension step was carried out for 10 min at 72°C. After completion of the PCR the total reaction mixture was spun down and mixed (3 *μ*L) before being loaded into the wells of a 1.2/1.8% agarose (Nacalai Tesque, Kyoto, Japan) gel. Electrophoresis was then performed for ~22 min at 100 volts in 1× TAE buffer using a Mupid‐ex electrophoresis system (ADVANCE, Tokyo, Japan). The gels were stained (8 *μ*L of 10 mg/mL ethidium bromide in 200 mL 1× TAE buffer) for ~7 min and the stained bands were visualized using an UV‐trans illuminator (ATTO, Tokyo, Japan).

### DNA microarray analysis

A rat 4 × 44K whole genome oligo DNA microarray chip (G4131F, Agilent Technologies, Palo Alto, CA) was used for global gene expression analysis using the hippocampi. Total RNA (900 ng; 300 ng each replicate pooled together) was labeled with either Cy3 or Cy5 dye using an Agilent Low RNA Input Fluorescent Linear Amplification Kit (Agilent Technologies). Fluorescently labeled targets of Sedentary, WR and RWR samples were hybridized to the same microarray slide with 60‐mer probes (Fig. 1C). A flip labeling (dye swap or reverse labeling with Cy3 and Cy5 dyes) procedure was followed to nullify the dye bias associated with unequal incorporation of the two Cy dyes into cDNA (Rosenzweig et al. 2004; Martin‐Magniette et al. 2005). The use of a dye‐swap approach provides a more stringent selection condition for changed gene expression profiling than the use of a simple single/two‐color approach (Hirano et al. 2009; Ogawa et al. 2011). Briefly, to select differentially expressed genes, we identified genes that were up regulated in chip 1 (Cy3/Cy5 label for sedentary and WR, respectively) but down regulated in chip 2 (Cy3/Cy5 label for WR and sedentary, respectively) for the hippocampi. The same selection criteria were applied for chips 3–4 (sedentary and RWR).

Hybridization and wash processes were performed according to the manufacturer's instructions, and hybridized microarrays were scanned using an Agilent microarray scanner, G2505C (Agilent Technologies). For the detection of significantly differentially expressed genes between each group, each slide image was processed by Agilent feature extraction software (ver 11.0.1.1). Briefly, (1) this program measured Cy3 and Cy5 signal intensities of whole probes; (2) dye‐bias tends to be signal intensity dependent, and therefore the software selected probes using a set by rank consistency filter for dye normalization; (3) normalization was performed by LOWESS (locally weighted linear regression), which calculates the log ratio of dye‐normalized Cy3 and Cy5 signals, as well as the final error of the log ratio; (4) the significance (*P*) value was based on the propagate error and universal error models; (5) the threshold of significance for differentially expressed genes was <0.01 (for the confidence that the feature was not differentially expressed); and (6) erroneous data generated owing to artifacts were eliminated before data analysis using the software. The outputs of microarray analysis used in this study will be made available under the series number GSE 52516 at the NCBI Gene Expression Omnibus (GEO) public functional genomics data repository (http://www.ncbi.nlm.nih.gov/geo/info/linking.html).

### Ingenuity pathways analysis (IPA)

The functional and network analyses were generated through the use of IPA (Ingenuity^®^ Systems, www.ingenuity.com). The data set from microarray, which is the differentially expressed (>/<1.5/0.75‐fold compared to sedentary) genes, and their corresponding fold change values were uploaded as an Excel spread sheet into the IPA tool. To create gene networks, genes were overlaid onto a global molecular network developed from information contained in the ingenuity knowledge base. The functional analysis identified the biological functions that were most significant to the data set (*P*‐value < 0.05) according to right‐tailed Fisher's exact test.

### Quantitative RT‐PCR

To validate gene changes in array data and test the BDNF signaling‐related molecules and expression of select transcripts were determined by quantitative real‐time PCR (PE‐ABI Prism 7300; Applied Biosystems, Foster City, CA) using a fast start universal SYBR green master mix (Roche Applied Science, Mannheim, Germany) following the protocol provided by the manufacturer. Primer3 software was used to design reaction primers and the sequences are listed in Table 1. In brief, after 10 min of incubation at 95°C, the PCR reaction was carried out for 40 cycles at 95°C for 15 sec, 60°C for 30 sec and 72°C for 30 sec. As a validated endogenous control, *β*‐actin was amplified in a separate reaction for normalization. Each sample was processed in duplicate and melting curve analysis was performed on all samples. To verify the quality of this RNA, the yield and purity were determined spectrophotometrically (IMPLEN, Germany) and visually confirmed using formaldehyde‐agarose gel electrophoresis.

**Table 1.** The list of primers used for real‐time PCR

Primer name	Direction	Sequence	NCBI accession no.
IL1b	Forward	5′‐ attgtggctgtggagaagct ‐3′	NM‐031512
	Reverse	5′‐ atgtcccgaccattgctgtt ‐3′	
IL2ra	Forward	5′‐ gcaacaactgtcagtgcaca ‐3′	NM‐013163
	Reverse	5′‐ tatgttgccaggtgaaccca ‐3′	
TNF	Forward	5′‐ gtgcctcagcctcttctcatt ‐3′	NM‐012675
	Reverse	5′‐ tcccaggtacatgggctcata ‐3′	
Cxcl1	Forward	5′‐ acagtggcagggattcactt ‐3′	NM‐030845
	Reverse	5′‐ tcgcgaccattcttgagtgt ‐3′	
Cxcl10	Forward	5′‐ atgaacccaagtgctgctgt ‐3′	NM‐139089
	Reverse	5′‐ tctttggctcaccgctttca ‐3′	
Ccl2	Forward	5′‐ aggtgtcccaaagaagctgt ‐3′	NM‐031530
	Reverse	5′‐ tgcttgaggtggttgtggaa ‐3′	
NFATc1	Forward	5′‐ tgtcgtgcagctacatggtt ‐3′	NM‐001244933
	Reverse	5′‐ tcggtcagttttcgcttcca ‐3′	
AVPr1a	Forward	5′‐ tttttgtggtggctgtgctg ‐3′	BC‐088095
	Reverse	5′‐ atgttgcgccagatgtggta ‐3′	
BDNF	Forward	5′‐ gcggcagataaaaagactgc ‐3′	NM‐012513
	Reverse	5′‐ gccagccaattctctttttg‐3′	
TrkB	Forward	5′‐ gacctgatcctgacgggtaa ‐3′	NM‐001163169
	Reverse	5′‐ tggtcacagacttcccttcc ‐3′	
CREB	Forward	5′‐ tcagccgggtactaccattc‐3′	X14788
	Reverse	5′‐ cctctctctttcgtgctgct ‐3′	
β‐Actin	Forward	5′‐ aaccctaaggccaaccgtga‐3′	NM‐031144
	Reverse	5′‐ cagggacaacacagcctgga ‐3′	

### Statistical analysis

Average running distance and work levels on each of the experimental days were analyzed with repeated measures two‐way ANOVA, followed by Bonferroni's multiple comparison test for post hoc analysis. The comparisons between different groups were analyzed with a one‐way ANOVA followed by Tukey's multiple comparison tests for post hoc analysis. Statistical significant difference was evaluated at *P *<**0.05.

## Results

### RWR increased work levels while decreasing running distance by half

Body weight decreased in both the WR and RWR groups compared with the sedentary group (*F*(2, 23) = 5.947, *P *<**0.05; Table 2). Repeated measures with ANOVA showed significance for day (*F*(27, 378) = 20.57, *P *<**0.0001), group (*F*(1, 14) = 9.40, *P *<**0.01) and interaction between day and group (*F*(27, 378) = 5.22, *P *<**0.0001) effects on daily running distance (Table 2). The average running distance of the RWR group (683.2 m/day) was significantly less than that of the WR group (1031.5 m/day) (*P *<**0.05; Table 2). The average work levels analysis revealed significance for day (*F*(27, 378) = 23.58, *P *<**0.0001), group (*F*(1, 14) = 32.79, *P *<**0.0001) and interaction between day and group (*F*(27, 378) = 20.43, *P *<**0.0001) effects (Table 2). The average work levels significantly increased by about 11‐fold in the RWR group (1247.9 N·m/kg b.w./day) compared to the WR group (112.1 N·m/kg b.w./day) (*P *<**0.01; Table 2).

**Table 2.** Effects of resistance wheel running (RWR) on body weight, exercise parameters, muscle adaptation, and stress‐related factors

	Group		
	Sedentary	WR	RWR
Body weight (g)	510.4 ± 8.4	457.6 ± 8.3**	462.5 ± 10.2*
Exercise parameters			
Average daily running distance (m)		1031.5 ± 42.2	683.2 ± 105.4*
Average daily work levels (N m/kg b.w./day)		112.1 ± 3.6	1247.9 ± 198.4***
Muscle adaptation			
Relative soleus wet mass to b.w. (mg/100 g)	45.2 ± 1.3	48.8 ± 1.5	50.5 ± 1.5*
Relative plantaris wet mass to b.w. (mg/100 g)	92.9 ± 1.6	101.5 ± 1.6	106.3 ± 3.9*
Stress‐related factors			
Relative adrenals Wt to b.w. (mg/100 g)	13.4 ± 0.5	14.2 ± 0.8	12.3 ± 0.8
Relative thymus Wt to b.w. (mg/100 g)	91.5 ± 5.6	105.1 ± 6.8	106.1 ± 4.9

All data are presented as the mean ± SE. BW, body weight; CS, citrate synthase; Wt, weight; Sed, sedentary; WR, wheel running with no resistance; RWR, resistance wheel running. Significant difference compared with sedentary after an ANOVA with Tukey's multiple comparison tests or Student's *t*‐test: *P <**0.05; ***P <**0.001.

### Fast‐twitch plantaris muscle adaptation by RWR without any negative stress effects

Hind limb skeletal muscle masses were analyzed to determine the effect of RWR on muscular adaptation post‐exercise regimen. Results showed that relative soleus muscle (*F*(2, 23) = 3.855, *P *<**0.05; Table 2) and plantaris muscle wet mass (*F*(2, 23) = 7.804, *P *<**0.01; Table 2) had increased only in the RWR group compared to the sedentary group. No significant change was found to the relative weights of adrenals (*F*(2, 23) = 1.783, *P *=**0.19) and thymus (*F*(2, 23) = 2.087, *P *=**0.14) (Table 2).

### RWR revealed down‐regulation as the dominant hippocampal gene expression profile

**Figure 2.**
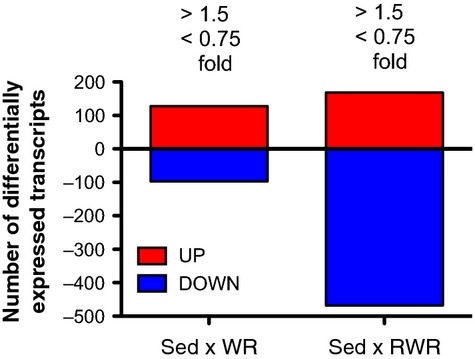
Differentially expressed genes in the WR and RWR. The numbers above each bar indicate the selection of genes from the total microarray datasets within a defined fold range of greater than 1.5‐fold and less than 0.75‐fold.

Genome‐wide global gene expression profiles were obtained for WR and RWR‐related genes. 128 (Sed × WR) and 169 (Sed × RWR) were up‐regulated (>1.5‐fold change) as compared with 97 (Sed × WR) and 468 (Sed × RWR) down‐regulated genes (<0.75‐fold change) after 4 weeks exercise, respectively (Fig. 2).

### Pathway and gene classification reveals differential gene expressions between RWR and WR

**Figure 3.**
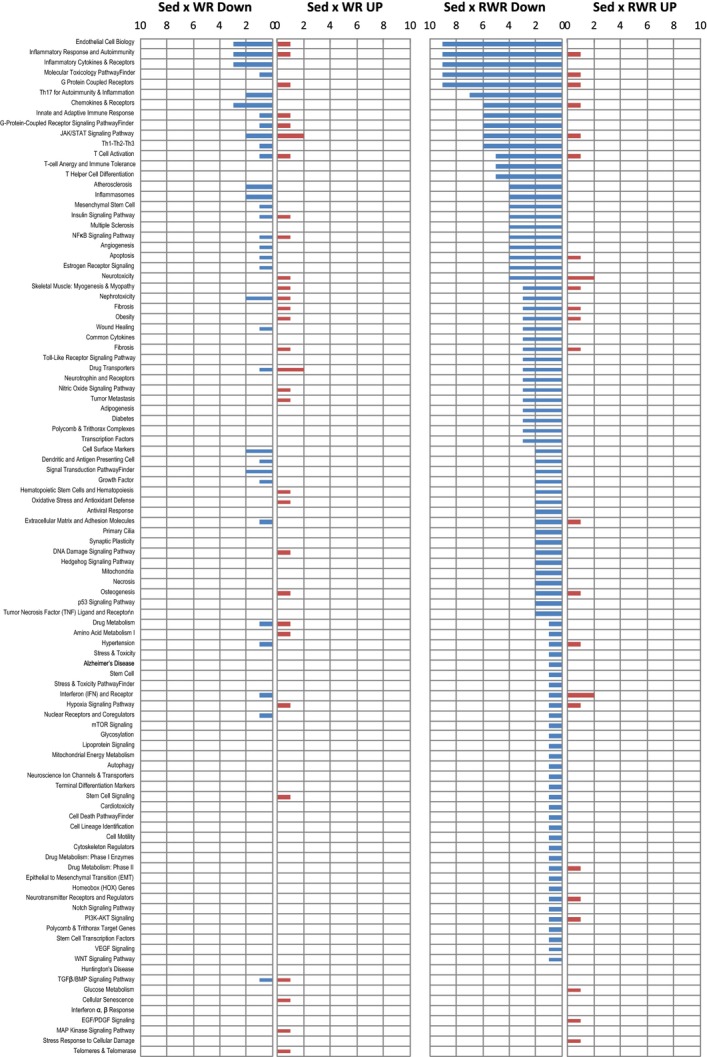
Biological pathway and gene classification. The up‐ and down‐regulated genes at Sed × WR and Sed × RWR were classified based on the available categories of more than 100 biological pathways and gene classification in the SABiosciences PCR array list. The numbers in the *y*‐axis represent number of genes in each category, which are indicated on the *x*‐axis.

The pathway‐focused gene classifications available on the QIAGEN website (SABiosciences; www.sabiosciences.com) were utilized to reveal the trend of predominant pathways affected in the hippocampus (Fig. 3). The up‐ and down‐regulated genes at sedentary × WR, and sedentary × RWR were classified based on the available categories of more than 100 biological pathways at hippocampus after 4 weeks of running. This categorization revealed that the trends of gene quantity (numbers) and quality (function) in the respective pathways varied between the two running modes.

### Biological function of genes by Ingenuity Pathway Analysis (IPA) unravels molecular networks influenced by RWR

**Figure 4.**
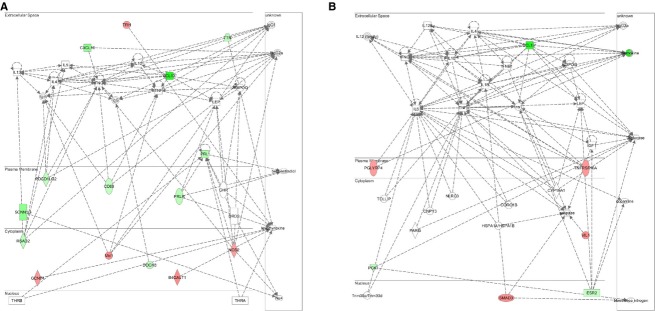
The two top gene networks sensitive to free wheel running. Ingenuity Pathway Analysis software was used to identify the most significantly modified gene networks from Sed × WR. (A) Network 1 is involved in Amino Acid Metabolism, Endocrine System Development and Function, Molecular Transport. (B) Network 2 is involved in Protein Synthesis, Gene Expression, Cellular Movement. Transcripts are color‐coded, according to expression changes (red, up‐regulation; green, down‐regulation). White indicates predicted molecules computational incorporated into networks based on evidence within the IPA knowledge base. Lines between molecules indicate a direct molecular connection.

**Figure 5.**
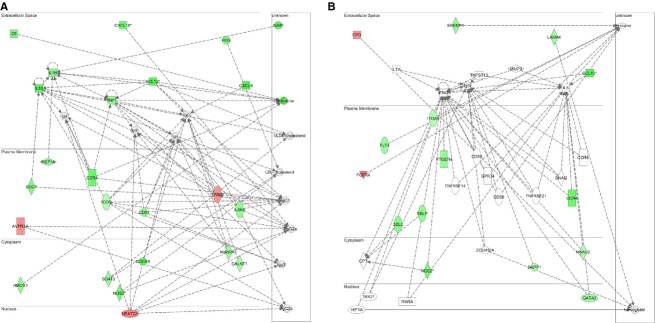
The two top gene networks sensitive to resistance wheel running. Ingenuity Pathway Analysis software was used to identify the most significantly modified gene networks from Sed × RWR. (A) Network 1 is involved in Humoral Immune Response, Protein Synthesis, Cellular Movement. (B) Network 2 is involved in Cellular Movement, Immune Cell Trafficking, Humoral Immune Response. Transcripts are color‐coded, according to expression changes (red, up‐regulation; green, down‐regulation). White indicates predicted molecules computational incorporated into networks based on evidence within the IPA knowledge base. Lines between molecules indicate a direct molecular connection.

Functional annotation of differentially expressed genes was performed using the IPA platform. The majority of these changes were unique to exercise mode (free wheel running or resistance wheel running), reflecting 4 weeks of the experiment. Table 3 lists the 10 most induced and repressed transcripts of the RWR group. In addition to this pathway, we analyzed the molecular connection between each hippocampal gene based on previous literature to gain further insight and to characterize the molecular behavior regulating hippocampal neuroplasticity at each intensity (Table 4). Networks were generated on the basis of known functions and interconnectivity of affected genes. The top five gene networks were constructed on the basis of the known functions and interconnectivity of altered genes (Table 5). WR‐regulated genes were involved in amino acid metabolism, endocrine system and molecular transport; however, RWR were involved in inflammatory/immune response, protein synthesis, and cellular movement. This was reflected in the top two modified molecular networks (Figs. 4, 5).

**Table 3.** The 10 hippocampal transcripts induced and 10 most repressed with resistance wheel running (RWR)

Symbol	Gene name	Agilent	Fold change	Location	Type(s)
Up‐regulated molecules					
NYX	Nyctalopin	A_44_P283938	3.000	Extracellular Space	Other
Cyp2c40 (includes others)	Cytochrome P450, family 2, subfamily c, polypeptide 40	A_44_P302721	2.830	Cytoplasm	Enzyme
PGK2	Phosphoglycerate kinase 2	A_44_P263049	2.760	Cytoplasm	Kinase
CDC6	Cell division cycle 6 homolog (*S. cerevisiae*)	A_44_P239281	2.440	Nucleus	Other
Cyp2b19	Cytochrome P450, family 2, subfamily b, polypeptide 19	A_44_P482563	2.140	Cytoplasm	Enzyme
FGFR4	Fibroblast growth factor receptor 4	A_44_P546866	2.090	Plasma Membrane	Kinase
Olfr1436/Olfr1437	Olfactory receptor 1437	A_44_P233666	2.090	Plasma Membrane	G‐protein‐coupled receptor
ANXA8L2 (includes others)	Annexin A8‐like 2	A_44_P525229	2.060	Plasma Membrane	Other
NCF2	Neutrophil cytosolic factor 2	A_44_P508554	2.060	Cytoplasm	Enzyme
CCKAR	Cholecystokinin A receptor	A_43_P15402	2.040	Plasma Membrane	G‐protein‐coupled receptor
Down‐regulated Molecules					
CXCL1	Chemokine (C‐X‐C motif) ligand 1	A_42_P473398	−4.348	Extracellular Space	Cytokine
PRL	Prolactin	A_43_P11492	−4.000	Extracellular Space	Cytokine
CXCL9	Chemokine (C‐X‐C motif) ligand 9	A_44_P1043157	−3.333	Extracellular Space	Cytokine
PDK4	Pyruvate dehydrogenase kinase, isozyme 4	A_44_P812772	−3.226	Cytoplasm	Kinase
CCL13	Chemokine (C‐C motif) ligand 13	A_42_P695401	−3.226	Extracellular Space	Cytokine
DOCK8	Dedicator of cytokinesis 8	A_44_P944006	−3.125	Cytoplasm	Other
OR6C1	Olfactory receptor, family 6, subfamily C, member 1	A_44_P974878	−3.030	Plasma Membrane	G‐protein‐coupled receptor
IL10	Interleukin 10	A_44_P384213	−3.030	Extracellular Space	Cytokine
ATP7B	ATPase, Cu++ transporting, beta polypeptide	A_44_P306339	−3.030	Cytoplasm	Transporter
TRPM1	Transient receptor potential cation channel, subfamily M, member 1	A_44_P295064	−2.941	Plasma Membrane	Ion channel

**Table 4.** Functional gene groupings sensitive to voluntary resistance wheel running (RWR)

Category	*P* value	Number of genes
Diseases and Disorders		
Immunological Disease	2.61E‐07–3.46E‐03	39
Hypersensitivity Response	7.24E‐07–1.38E‐03	19
Inflammatory Response	2.28E‐06–3.89E‐03	49
Cardiovascular Disease	3.25E‐06–2.44E‐03	59
Infectious Disease	4.87E‐06–3.12E‐03	31
Molecular and Cellular Functions		
Protein Synthesis	8.75E‐09–3.60E‐03	26
Cellular Movement	3.78E‐07–3.78E‐03	41
Cell‐To‐Cell Signaling and Interaction	1.61E‐06–3.89E‐03	50
Cell Signaling	7.76E‐06–2.67E‐03	38
Small Molecule Biochemistry	7.76E‐06–3.60E‐03	40
Physiological System Development and Function		
Nervous System Development and Function	3.93E‐11–3.00E‐03	73
Humoral Immune Response	8.75E‐09–3.46E‐03	24
Cell‐mediated Immune Response	3.78E‐07–3.88E‐03	24
Hematological System Development and Function	3.78E‐07–3.89E‐03	68
Immune Cell Trafficking	3.78E‐07–3.89E‐03	46

Functional groupings of transcripts differentially modified by RWR (also shown are *P*‐values, and numbers of involved genes). The groups derived from Ingenuity Pathway Analysis (IPA) are categorized into disease and disorders, molecular functions, and physiological system development and function.

**Table 5.** The top five gene networks modified with wheel running (WR) and resistance wheel running (RWR)

Network	Associated network functions	Score	Focus molecules	Molecules in network
Sedentary × WR				
1	Amino Acid Metabolism, Endocrine System Development and Function, Molecular Transport	30	15	ADIPOQ,B4GALT1,beta‐stradiol,CCL13,CD69,CXCL10,DOCK8,DRD2,GCNT4,GHR,IFNG,IgG1,IgG,IgG2a,Igm,IL4,IL5,IL10,IL13,LEP,levothyroxine,Mbl1,Na+,NOS2, PDCD1LG2,PRL,PRLR,RSAD2,SCNN1G,THRA,THRB,TNF,TRH,TTR2
2	Protein Synthesis, Gene Expression, Cellular Movement	11	7	ADIPOQ,blood urea nitrogen,caspase,CCL13,chemokine,CNPY3,CORO1B,CYP19A1,D‐glucose,dopamine,ESR2,HSPA1A/HSPA1B,IFNB1,IFNG,IGF1,IgG2a,IL4,IL6,IL10,IL12 (family),IL12B,IL1B,Ins1,LEP,NLRC3,PARG,PCK1,PGLYRP4,SMAD3,TNF,TNFRSF10A,TOLLIP,Trim30a/Trim30d,VIL1
3	Cellular Movement	2	1	Mapk, PIK3R6
4	Cell Morphology, Connective Tissue Development and Function, Embryonic Development	2	1	GPR55,PLC
5	Drug Metabolism, Lipid Metabolism, Molecular Transport	2	1	ADH1C,ethanol
Sedentary × RWR				
1	Humoral Immune Response, Protein Synthesis, Cellular Movement	44	24	AVPR1A,C5,CCL13,CCR4,CD81,chemokine,CXCL9,CXCL10,DOCK8,FGG,GALNT1, HMOX1,ICOS,Iga,Ige,IgG1,Igg3,IgG,IgG2a,IgG2b,Igm,IL10,IL1B,IL2RA, LDL‐cholesterol,MAN2A1,MEP1A,NAIP,NFATC1,NOS2,SDC1,SOAT2,TNF,TRB@, VLDL‐cholesterol
2	Cellular Movement, Immune Cell Trafficking, Humoral Immune Response	23	15	CCL13,CCR4,CCR6,CD69,CD80,DAPP1,EFEMP1,EPO,FGFR4,FLT4,GATA3,GNAI2, GPR34,GPT,hemoglobin,HIF1A,IFNG,Igm,IL5,ITGA5,L‐tyrosine,LAMA4,LTA,MMP9, NOS2,PTGER4,RARA,RSAD2,SELL,SELP,TBX21,TNFRSF14,TNFRSF21,TNFSF13, ZC3H12A
3	Protein Synthesis, Endocrine System Development and Function, Molecular Transport	17	12	ABCA1,ADCYAP1,ALB,APOE,beta‐estradiol,CACNA1C,CCKAR,CD36,CGA, CYP19A1,DRD2,ESR1,FSH,GABBR1,GCG,GHR,GPR119,HDL‐cholesterol, Immunoglobulin,Insulin,IRS1,IRS2,LATS1,Lh,NOS2,PGR,PRL,PRLR,progesterone, SST,SYT9,testosterone,THRA,THRB
4	Carbohydrate Metabolism, Molecular Transport, Small Molecule Biochemistry	17	12	AIM2,AMELX,CCL13,CKM,Collagen(s),D‐glucose,ERC1,glycogen,H6PD, IHH,IKBKG,Ins1,IRAK2,IRAK3,IRS2,IRS4,NFkB (complex),NFKBIB,NOD1, PARG,PDK4,PGR,RHEBL1,RIPK1,RIPK2,RPS6KA4,RPS6KA5, SLC5A1,TGFB1,THRA,TNF,TNFRSF8,TNFSF14,TRAF2,UCP3
5	Cell Death and Survival, Cancer, Hematological Disease	10	5	BCL2L1, caspase,COL4A3,FASLG,PML,TNFAIP8

The “Score” reflects the negative log of the *P* value and signifies the likelihood of network‐eligible genes within a network being clustered together as a result of chance. See (Figs. 4, 5) for details of networks 1 and 2.

### Confirmation of gene expression by RT‐PCR and differential expression of their mRNAs in the hippocampus

**Figure 6.**
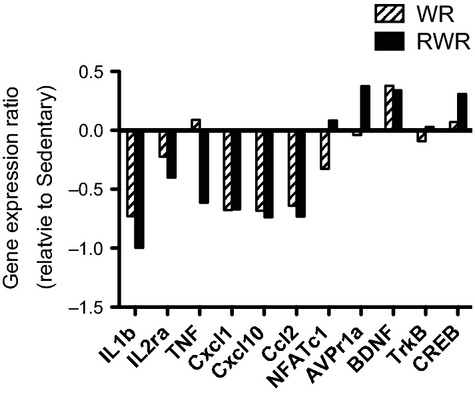
Confirmation of differentially modified transcripts form microarray analysis. BDNF signalling‐related genes and most up‐ or down‐regulated by WR or RWR were selected for validation via quantitative real‐time PCR. Each column indicates WR (diagonal stripes columns) or RWR (black columns), respectively. For each gene, the expression change is represented as the fold change in WR or RWR relative to Sedentary.

To validate gene changes in DNA microarray data and test the robustness of these responses, quantitative RT‐PCR was used to assess expression of select transcripts. We selected 11 genes with annotated functions that were highly up‐regulated or down‐regulated at the sedentary x RWR condition. As a result of their potential relevance to cognitive functions, transcripts involved in BDNF signaling were also analyzed. The mRNA expression profiles revealed that the DNA microarray data could be validated using appropriate primer design (Fig. 6).

## Discussion

To identify gene expression changes, the hippocampi from adult rats were dissected after 4 weeks of wheel running with (RWR) and without (WR) a load. Our results indicated that voluntary RWR increased the work levels by about 11‐fold but decreased running distance, which resulted in the muscular adaptation of fast‐twitch plantaris muscle without negative stress effects, validating RWR as a strength training model. Quantitatively comparing the gene expression profile in exercised rats with sedentary controls, it was seen that RWR recruited a more varied set of transcriptional categories, which were predominantly suppressed or down‐regulated. These categories included transcripts involved in humoral immune response, protein synthesis, and cellular movement. Among the genes specifically regulated with RWR, *NFATc1, AVPR1A*, and *FGFR4* were newly identified factors in response to exercise. RWR also highly down‐regulated inflammatory cytokines (*IL1B*,* IL2RA*, and *TNF*) and chemokines (*CXCL1*,* CXCL10*,* CCL2*, and *CCR4*), along with *SYCP3*,* PRL* genes. This study suggests new information on voluntary RWR‐influenced transcriptome in rat hippocampus, and that these gene candidates likely play a more critical role in the development of greater hippocampal adaptations with RWR than with WR.

### RWR is an excellent model for muscular and hippocampal adaptations

An important characteristic of voluntary resistance wheel running (RWR) is that it allows for a given load on a running wheel (range of 0–200 g) and for increasing work levels, which results in muscular adaptation in fast‐twitch muscle without physical and psychological stressors such as electrical shock and additional weight (Ishihara et al. 1998; Call et al. 2010). RWR differs from forced treadmill running, swimming or ladder climbing because with RWR animals exercise of their own volition. In addition, the wheel running represents intermittent, nonexhaustive, energy‐efficient activity (De Bono et al. 2006; Leasure and Jones 2008), and stress levels are demonstrably lower in voluntary exercise compared to forced exercise (Moraska et al. 2000; Ke et al. 2011). Thus, RWR activity should reflect greater contributions of the activity per se versus the emotional or neurohumoral stresses associated with forced exercise. Indeed, our recently concluded studies and the present results show that RWR, with shorter distances but higher work levels, elicits muscular adaptation for fast‐twitch plantaris muscle without causing negative chronic stress effects (Lee et al. 2012, 2013), which is also in agreement with previous findings (Ishihara et al. 1998; Konhilas et al. 2005; Legerlotz et al. 2008). Our data also provide evidence to show that RWR enhances spatial memory associated with hippocampal BDNF signaling and hippocampal neurogenesis (Lee et al. 2012, 2013). In all, RWR represents a successful model that enhances the effects of exercise on both muscular and hippocampal adaptations, even with short distances.

### RWR influences gene down‐regulation as the dominant expression profile, and which is associated with hippocampal plasticity

As discussed above, RWR is an excellent model for both muscular and hippocampal adaptations, leading us to hypothesize that resistance wheel running would produce positive changes at the level of transcription within the hippocampus. Uncovering these novel gene functions/candidates, using a DNA microarray approach might be helpful in understanding the underlying mechanisms for hippocampal plasticity. As a first step, we classified genes that were dramatically up‐regulated or down‐regulated following 4 weeks of wheel running. Surprisingly, RWR recruited a more varied set of transcriptional categories that was observed to be predominantly suppressed or down‐regulated (Fig. 2, Table 3). First, we discuss below some of the identified noticeable gene alterations according to their respective functions.

### Change in gene expressions with RWR in gene classification and Ingenuity Pathway Analysis (IPA)

To reveal the trend of predominant pathways and the most significantly modified genes affected by RWR, we further utilized pathway‐focused or specific‐gene classification and Ingenuity Pathway Analysis (IPA). In the present study, most pathways were dominated by transcriptional down‐regulation (Fig. 3). Down‐regulated gene functions in RWR are associated with the major categories of endothelial cell biology, inflammatory response and autoimmunity, inflammatory cytokines & receptors, molecular toxicology and pathway finder, and G protein‐coupled receptors. It should be noted that the same genes could be included in different categories. In addition, IPA analysis revealed that genes related to humoral immune response, protein synthesis, cellular movement, and immune cell trafficking strongly changed expression in the RWR group (Fig. 5A and B).

### Up‐regulated genes involved in hippocampal plasticity

Among the genes specifically regulated genes by RWR, we found an increased expression of novel molecules in the hippocampus, which are being reported for the first time in this paper. These include hydrolethalus syndrome protein 1 (*HYLS1*), nyctalopin (*NYX*), phosphoglycerate kinase 2 *(PGK2*), nuclear factor of activated T cells, cytoplasmic 1 (*NFATc1*), arginine vasopressin receptor 1A (*AVPR1A*), and fibroblast growth factor receptor 4 (*FGFR4*), all showing a marked up‐regulation in the hippocampus as a result of RWR. In particular, the transcription factor *NFAT* has been shown to be an important regulator in the proliferation and differentiation of neural development and plasticity (Graef et al. 1999; Schwartz et al. 2009). And, the *AVPR1A* gene is expressed widely in the brain where it modulates a range of behaviors from learning, memory, and responses to stressors to social behaviors (Bielsky et al. 2004; Caldwell et al. 2008). Moreover, the *FGF* responds to adult neurogenesis by increasing proliferation, differentiation, and survival (Galvez‐Contreras et al. 2012). Therefore, further studies are needed to verify whether the RWR‐induced increase in these novel molecules is indeed related to hippocampal plasticity.

### Down‐regulated genes involved in hippocampal plasticity

We found a greatly down‐regulated *SYCP3* gene encoding the synaptonemal complex protein 3, which is a marker for cell transformation that has prognostic significance in various cancers. *SYCP3* is a DNA‐binding protein and a structural component of the synaptonemal complex (Yuan et al. 1998), and *SYCP3*‐mediated PI3K/Akt activation appears to be highly associated with resistance of tumor cells to cancer drugs (Noh et al. 2009). Moreover, inhibition of PI3K/Akt signaling blocks exercise‐mediated enhancement of neurogenesis and synaptic plasticity in the hippocampus (Bruel‐Jungerman et al. 2009). Therefore, *SYCP3*‐mediated PI3K/Akt activation has been shown to play a significant role *via* anti‐apoptotic function in promoting survival of newly formed granule cells generated during exercise and the associated increase in synaptic plasticity in the dentate gyrus.

Another notable gene was prolactin (*PRL*) that was also highly down‐regulated with RWR. *PRL*, primarily known for its hormonal actions, has recently been identified as a neuropeptide of the brain. A variety of behavioral and neuronal actions have been described to be regulated by brain *PRL* (Ben‐Jonathan et al. 1996). Such effects include anxiolytics in male and female rats (Torner et al. 2001), attenuation of hormonal and neuronal responses to various stressors (Donner et al. 2007), and regulation of maternal behavior (Bridges et al. 1990). Interestingly, trophic actions of *PRL* in the central nervous system include mediating development and maturation of dopaminergic neurons, regulating neurogenesis (Furuta and Bridges 2005), and brain cell proliferation (Mangoura et al. 2000). Prolactin prevents chronic stress‐induced decrease of adult hippocampal neurogenesis and promotes a positive neuronal cell fate (Torner et al. 2009). These results indicate that *PRL* protects neurogenesis in the DG of chronically stressed mice.

### Down‐regulated inflammatory cytokines and chemokines

We have focused our attention on the genes of inflammatory cytokines and chemokines, and the expression of numerous chemokine‐related genes, including genes encoding for chemokine (C‐x‐C motif and C‐C motif) ligands that were highly down‐regulated by RWR. Our data showed RWR down‐regulated pro‐inflammatory cytokines (*IL1B, IL2RA, TNF*) levels in the hippocampus, findings which are consistent with previous reports that physical activity led to decreases of proinflammatory or immunity‐related genes (Petersen and Pedersen 2005; Bronikowski et al. 2003; Chennaoui et al. 2008; Gomes da Silva et al. 2013). It is important to point out that a proinflammatory cytokines induction in the brain might result in neuronal dysfunction and vulnerability. Indeed, increased levels of proinflammatory cytokines have been demonstrated to disturb neurogenesis (Ekdahl et al. 2003) and cognitive function (Arai et al. 2001). Moreover, higher levels of inflammatory cytokines are reported in the aged brain (Godbout and Johnson 2006) and neurodegenerative disorders such as Alzheimer's disease and other chronic conditions (Lucas et al. 2006). The importance of inflammatory response induced by exercise indicates its potential therapeutic potential for exercise‐related brain inflammatory imbalance as well as to its potential to reduce the risk of neuro‐inflammation‐linked disorders, and this plays a crucial role in preventing damaged neurons from undergoing apoptosis (Rosczyk et al. 2008). Thus, these findings indicate a favorable effect of RWR on the hippocampal pro‐ and anti‐inflammatory balance of the rats, and provide information of potential interest in showing the involvement of numerous interleukin family members in exercised‐induced hippocampal plasticity.

Another example was of the inflammatory cytokines, which are also known to be important in hippocampus‐dependent functions such as spatial learning and memory (Sparkman et al. 2005). Our data showed that *CXCL1* was the most highly down‐regulated gene (−4.34 fold) with RWR. We also identified *CXCL9*,* CXCL10*,* CCL2*,* CCL13* and *CCR4* as being down‐regulated following 4 weeks of RWR. These results reveal the differential and specific regulation of the Cxcl‐ and Ccl‐family members in the hippocampus. The chemokines are chemotactic cytokines that, together with their receptors expressed on leukocytes, play crucial roles in the extravasation and migration of leukocytes under inflammatory conditions (Yamakoshi et al. 2007; Donde et al. 2012). In addition, inflammation actions disturb hippocampal neurogenesis related to the performance of spatial memory tasks (Ekdahl et al. 2003; Monje et al. 2003). Therefore, these results suggest that chemokine with important roles in the hippocampus could affect normal brain functions and vulnerability.

### Confirmation of gene expression by quantitative RT‐PCR

Several cellular and molecular systems important for maintaining neuronal function and plasticity, such as neurotrophins may be involved in positive effects of exercise on the brain. In the present study, we found that RWR increased both hippocampal BDNF, and p‐CREB mRNA levels by quantitative RT‐PCR (Fig. 6). It is likely that the potential roles of RWR in hippocampal function are associated with the action of BDNF signaling by integrating novel gene mechanisms of synaptic plasticity. However, we do not currently have proof for the above hypothesis. Nonetheless, our study provides information on unique expressed genes whose further analysis might shed light on their role in the RWR hippocampus.

### Conclusion and prospect

Here, we fulfill the study objectives by (1) providing an inventory of newly changed gene expression in exercised rat hippocampi; (2) identifying alterations in a number of transcriptional pathways implicated in plasticity; and (3) unraveling specific transcripts altered in RWR compared with the WR that might be crucial to the enhancement of hippocampal functions. This first study adopting a whole‐genome DNA microarray analysis approach has screened molecular factors underpinning the benefits of RWR. We speculate that the identified unique gene candidates will form part of our future investigations on the effects of target neuropeptides in potentially RWR‐induced improvement of hippocampal functions. These future studies will provide new concepts of the sites of gene expression and increase our understanding of the crucial functional role of various genes in response to RWR, short distances but high work levels, for effective exercise leading to nonpharmacological hippocampal plasticity.

## Conflict of Interest

No conflicts of interests, financial or otherwise, are declared by the author(s).
